# Supplementary material for: Rab41-mediated ESCRT machinery repairs membrane rupture by a bacterial toxin in xenophagy
Source: Nat Commun. 2023 Oct 6;14:6230. doi: 10.1038/s41467-023-42039-2 (PMC10558455; doi:10.1038/s41467-023-42039-2)
Supplement: Supplementary file 1 — Supplementary Information [file 41467_2023_42039_MOESM1_ESM.pdf]

SUPPLEMENTARY FIGURES

Rab41-mediated ESCRT machinery repairs membrane rupture by a bacterial toxin in xenophagy

Takashi Nozawa[1], Hirotaka Toh[1], Junpei Iibushi[1], Kohei Kogai[1], Atsuko Minowa-Nozawa[1], Junko Satoh[2], Shinji Ito[2], Kazunori Murase[1], and Ichiro Nakagawa[1,*]

[1]Department of Microbiology, Graduate School of Medicine, Kyoto University, Yoshida-Konoe-cho, Sakyo-ku, Kyoto 606-8501, Japan
[2] Medical Research Support Center, Graduate School of Medicine, Kyoto University, Yoshida-Konoe-cho, Sakyo-ku, Kyoto 606-8501, Japan

*Correspondence: nakagawa.ichiro.7w@kyoto-u.ac.jp (I.N.)

a

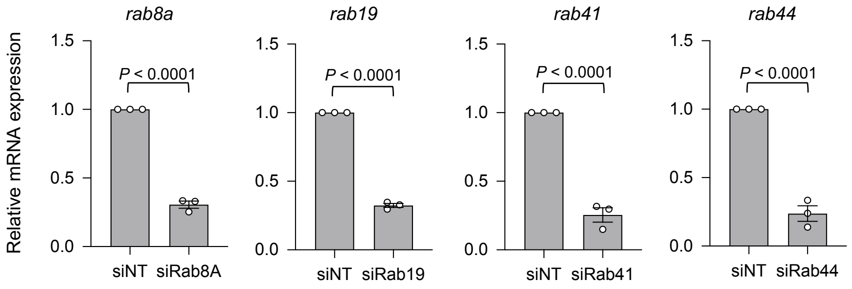

b

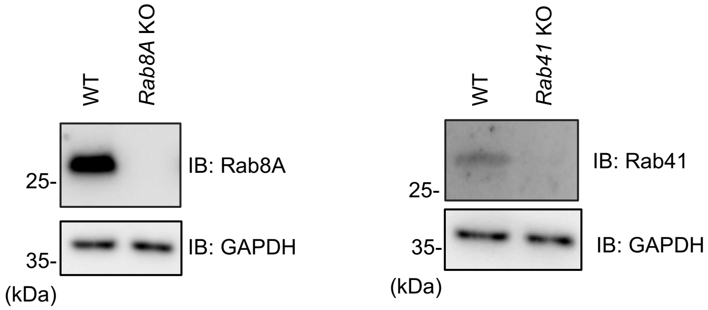

Supplementary Fig. 1 Effects of knockdown or knockdown of Rab8A and Rab41

a HeLa cells were transfected with indicated siRNA. Forty eight hour after transfection, the mRNA expression of indicated Rabs were detected by qPCR. Data are individual values and mean ± SEM (n = 3 biologically independent experiments) . Unpaired two-tailed *t* test.

b Immunoblot analysis of *Rab8A* or *Rab41* knockout cells. Shown are representative immunoblot images of three independent experiments. Source data are provided as a Source Data file.

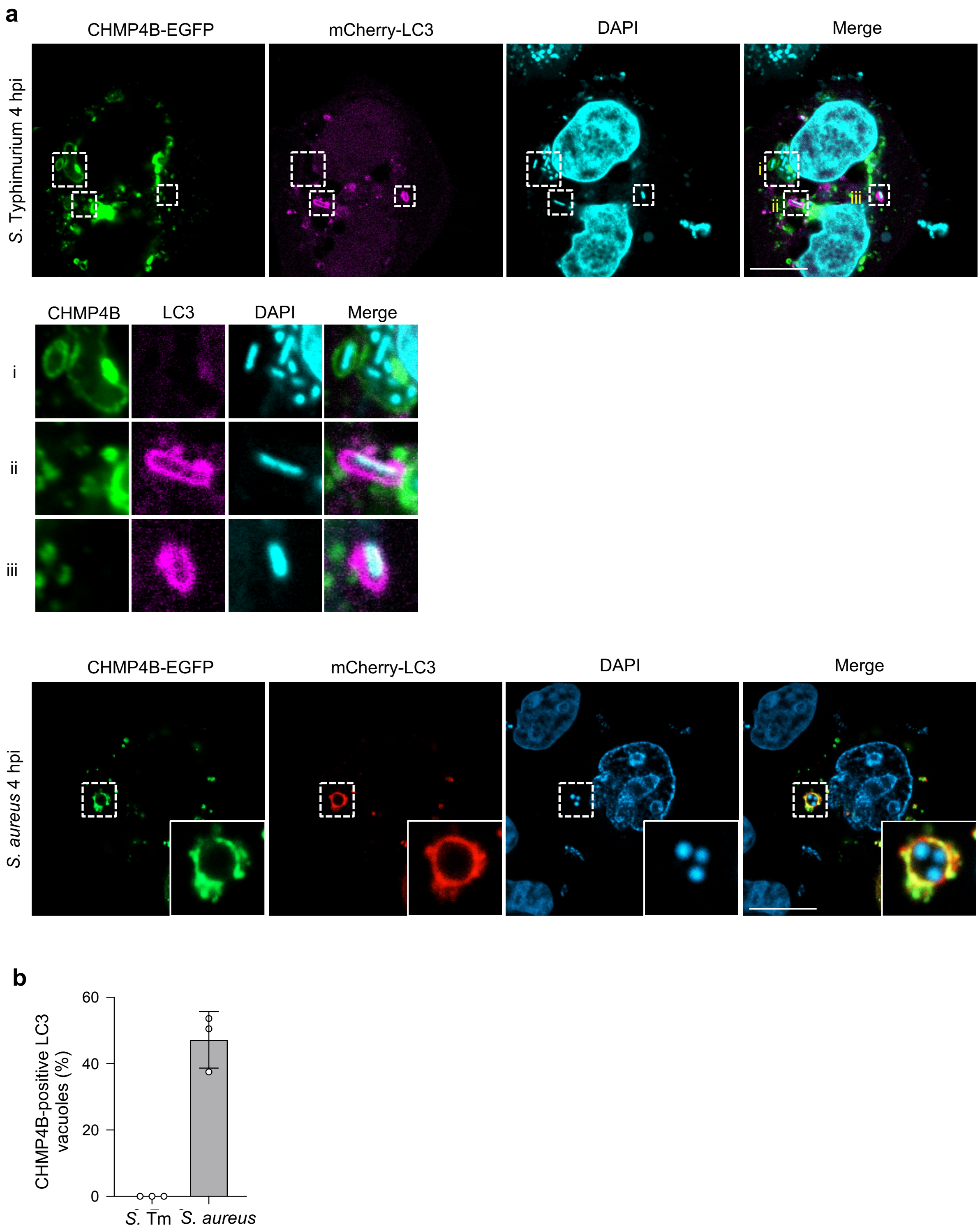

Supplementary Fig. 2 ESCRT-III localization during *S.* Typhimurium or *S. aureus* infection
a, b HeLa cells expressing CHMP4B-EGFP and mCherry-LC3 were infected with *S.* Typhimurium or *S. aureus* for 4 h. Cellular and bacterial DNAs were stained with DAPI. Shown are confocal single-slice images of three independent experiments (**a**), and quantification of CHMP4B-positive bacteria-containing LC3 vacuoles (BcLV) (**b**). Scale bar, 10 μm. Data are individual values and mean ± SEM from 30 BcLV analyzed per experiment, and three independent experiments were performed. Source data are provided as a Source Data file.

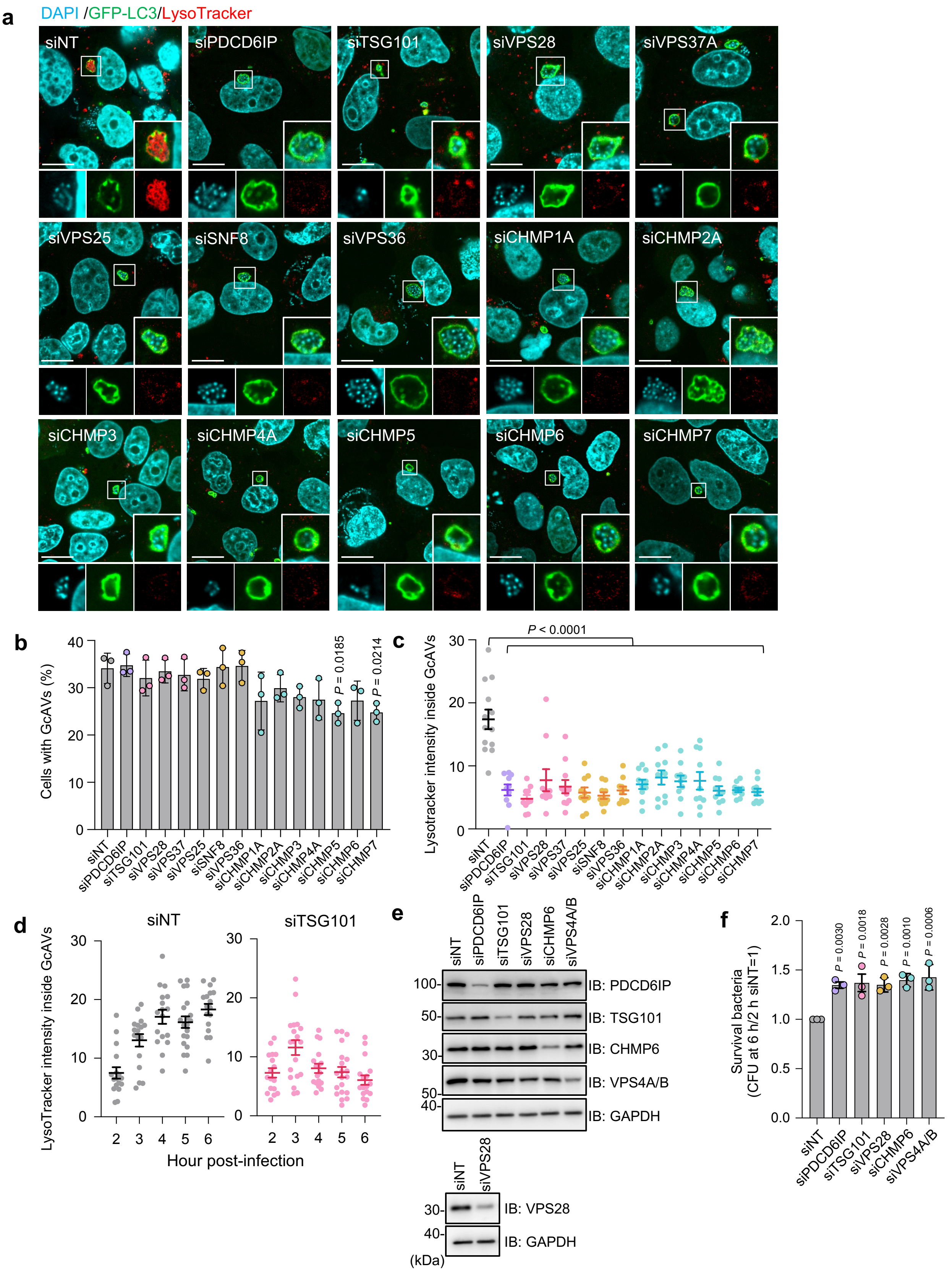

Supplementary Fig. 3 ESCRT proteins are required for maintaining xenophagosome acidification and bacterial degradation

a, b, c HeLa cells stably expressing GFP-LC3 were transfected with indicated siRNAs, infected with GAS for 4 h. Cells were incubated with LysoTracker Red for 30 min prior to the fixation. Cellular and bacterial DNAs were stained with DAPI. Shown are representative confocal single-slice images of three independent experiments (**a**), quantification of cells with GcVAs (**b**), and LysoTracker intensity within GcAVs (**c**). Scale bar, 10 μm. Data in (**b**) are individual values and mean ± SEM (n = 3 biologically independent experiments and 200 < cells were examined in each experiment). Data in (**C**) are individual values and mean ± SEM (n = 10 GcAVs examined over 3 independent experiments). One-way ANOVA, Dunnett's test.

d Time course analysis of GcAV acidification in TSG101-knockdown cells. Data are individual values and mean ± SEM (n = 19 GcAVs examined over 3 independent experiments).

e Immunoblotting of siRNA-transfected cells. HeLa cells were transfected with indicated siRNA oligonucleotides for 48 h, and cell lysate were analyzed by immunoblotting using specific antibodies. Shown are representative immunoblot images of three independent experiments.

f Intracellular bacterial CFU ratio at 6 hpi/2 hpi in the control and ESCRT proteins-knockdown cells. Data are individual values and mean ± SEM (n = 3 biologically independent experiments) . One-way ANOVA, Dunnett's test.

Source data are provided as a Source Data file.

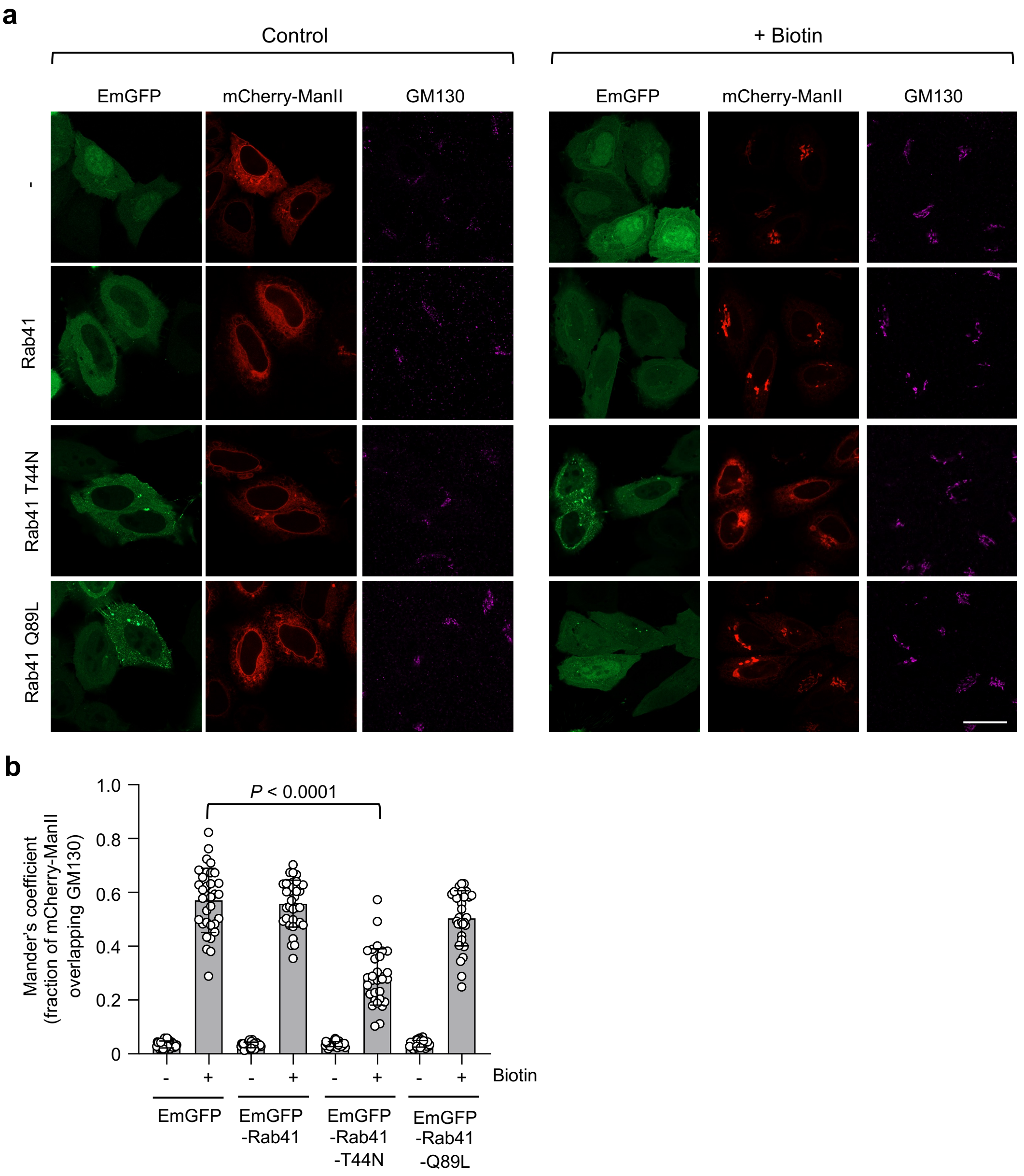

Supplementary Fig. 4 Rab41 is involved in the ER to the Golgi trafficking in a GTPase-dependent manner
a HeLa cells expressing streptavidin-KDEL and SBP-mCherry-ManII, and indicated EmGFP-Rab41 constructs were incubated for 1 h with biotin to observe the traffic of SBP-mCherry-ManII from the ER to the Golgi apparatus. Cells were then fixed, and immunostained for endogenous GM130. Shown are represennative confocal single-slice images of three independent experiments. Scale bars, 10 μm.
b Quantification of the proportions of reporter (mCherry-ManII) overlapping GM130 from 30 cells shown in (**a**). One-way ANOVA, Tukey's test.
Source data are provided as a Source Data file.

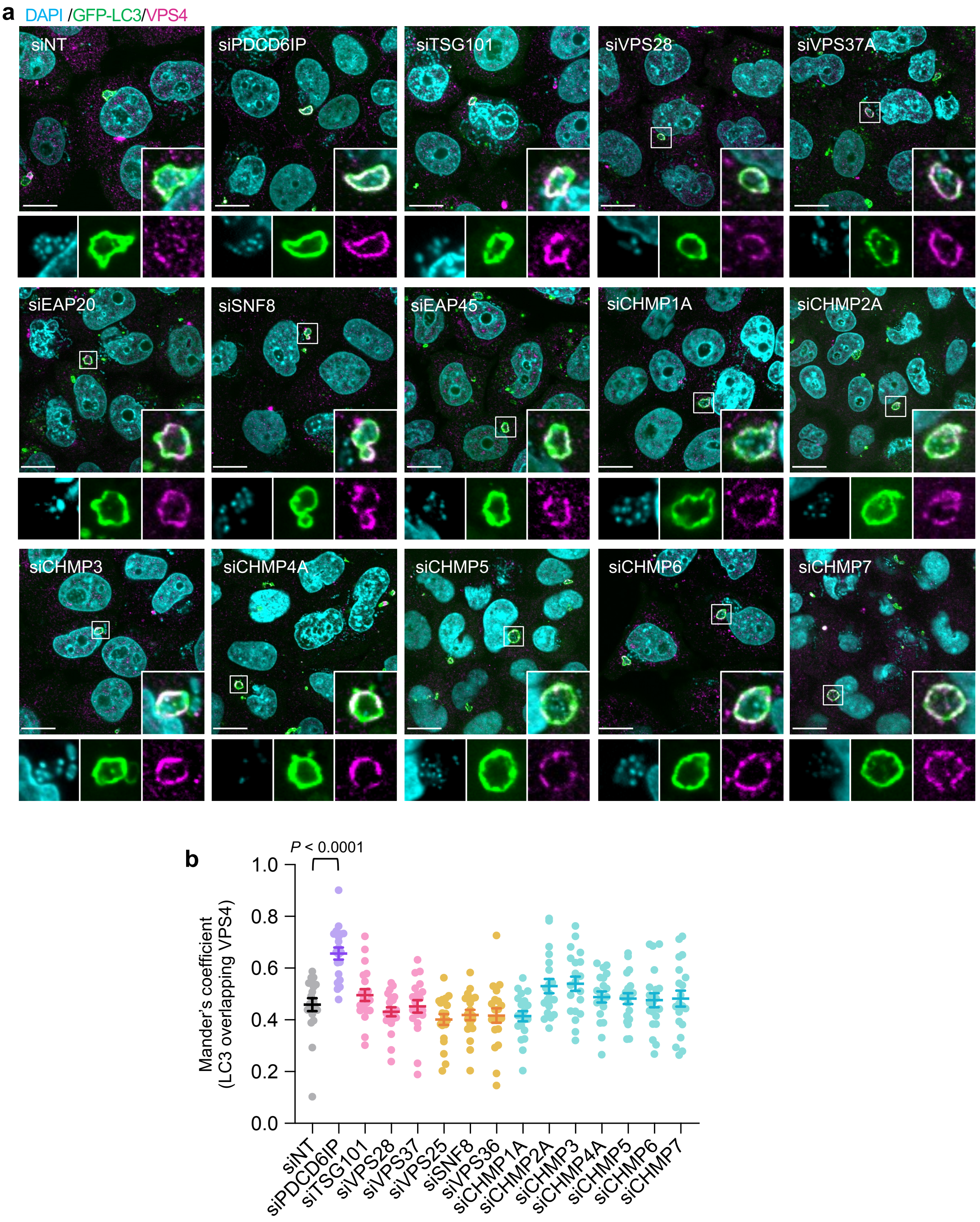

Supplementary Fig. 5 Effects of knockdown of ESCRT components on the recruitment of VPS4 to GcAVs

a, b HeLa cells stably expressing GFP-LC3 were transfected with indicated siRNAs, infected with GAS for 4 h. Cells were fixed, immunostained for endogenous VPS4, and stained with DAPI. Shown are confocal single-slice representative images of three independent experiments. Scale bars, 10 μm. Quantification of LC3 signals (GcAVs) overlapping VPS4 from 20 GcAVs shown in (**a**) (**b**). Data in (**b**) are individual values and mean ± SEM (n = 20 GcAVs examined over three independent experiments). One-way ANOVA, Dunnett's test.

Source data are provided as a Source Data file.

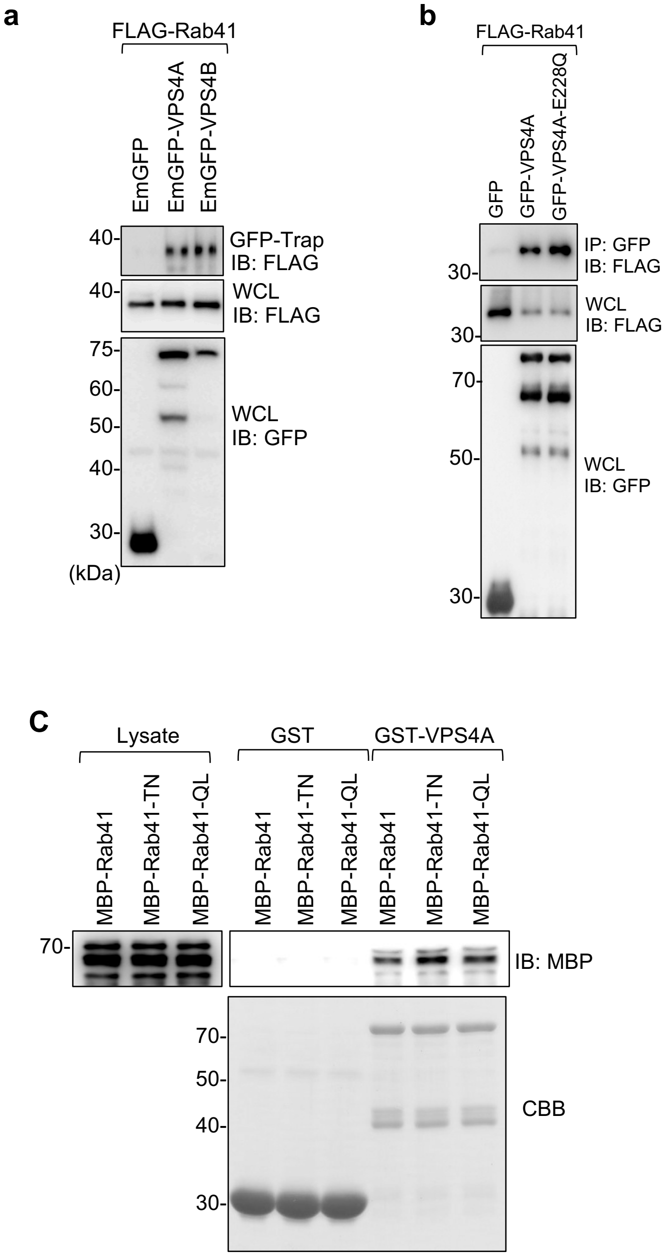

Supplementary Fig. 6 Interaction between Rab41 and VPS4

a Co-immunoprecipitation analysis. HEK293T cells expressing FLAG-Rab41 and EmGFP, EmGFP-VPS4A, or EmGFP-VPS4B were lysed, then the lysates were immunoprecipitated with GFP-Trap beads. The resulting samples were analyzed by immunoblot analysis. Data shown are representative of three independent experiments.

b Co-immunoprecipitation analysis. HEK293T cells expressing FLAG-Rab41 and EmGFP, EmGFP-VPS4A, or EmGFP-VPS4A-E228Q were lysed, then the lysates were immunoprecipitated with GFP-Trap beads. The resulting samples were analyzed by immunoblot analysis. Data shown are representative of three independent experiments.

c Pulldown of GST-tagged VPS4 with MBP-Rab41, Rab41-T44N or Rab41-Q89L expressed in *E. coli*. The resulting samples were analyzed by immunoblot analysis or CBB staining. Data shown are representative of three independent experiments. CBB, coomassie brilliant blue.

Source data are provided as a Source Data file.

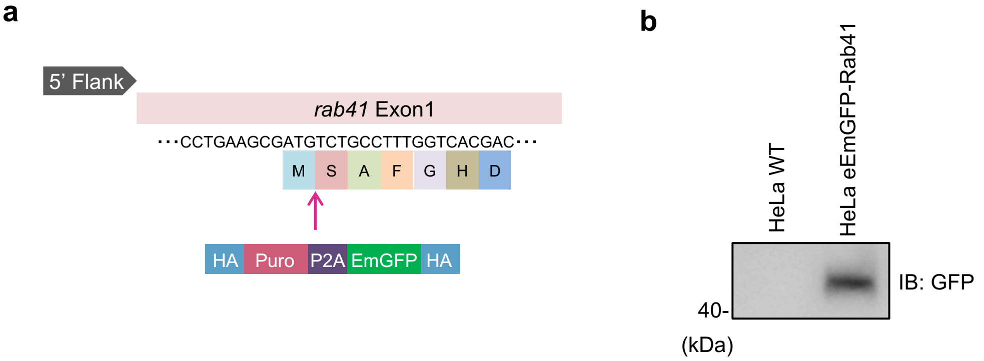

Supplementary Fig. 7 Generation of EmGFP-Rab41 knockin HeLa cells

a Schematics of the insertion of EmGFP into the N-terminus of Rab41. Insertion of donor DNA including homologous arm (HA), puromycin-resistant gene, P2A, and EmGFP to the site immediately after the start ATG.

b Immunoblot analysis to detect endogenously expressed EmGFP-Rab41. Data shown is representative of three independent experiments.

Source data are provided as a Source Data file.

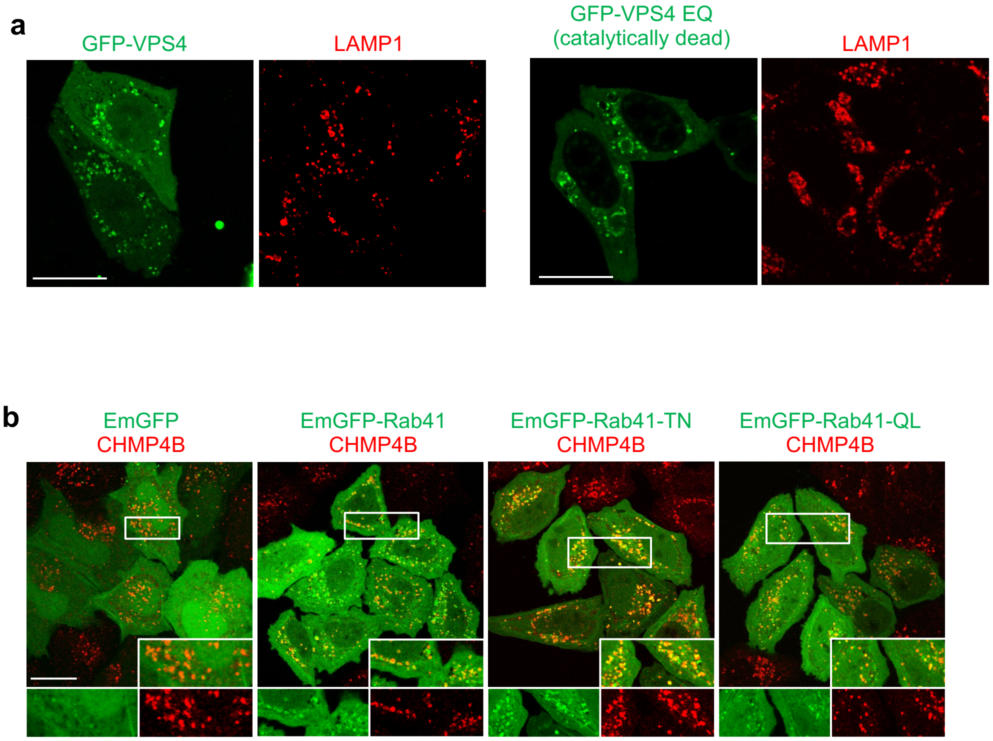

Supplementary Fig. 8 Effects of VPS4 and Rab41 mutants expression on lysosomal morphology

a HeLa cells expressing GFP-VPS4 or GFP-VPS4 E228Q (ATPase deficient mutant) were immunostained for endogenous LAMP1. Shown are representative confocal single-slice images of three independent experiments.

b HeLa cells expressing EmGFP or EmGFP-Rab41 mutants were treated with LLOMe for 1 h, fixed and immunostained for endogenous CHMP4B. Shown are representative confocal single-slice images of three independent experiments. Bars; 10 μm.

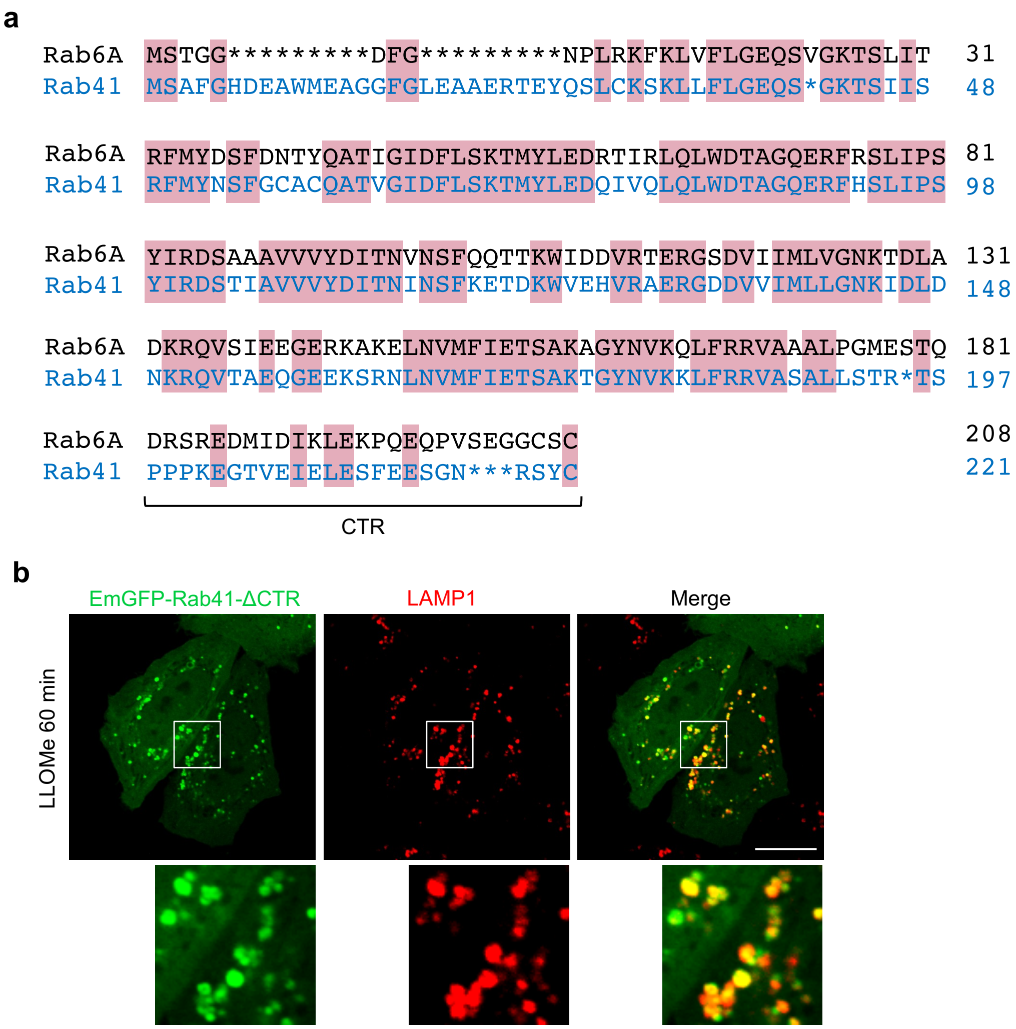

Supplementary Fig. 9 Localization of Rab41 CTR deletion mutants

a Alignment of amino acid sequences of Rab6A and Rab41 (Rab6D). Multiple sequence alignment was performed using T-COFFEE[68]. Consensus sequences were shown with red background. CTR, C terminal region.

b HeLa cells expressing EmGFP-Rab41 C-terminal region (CTR) deletion mutant were treated with LLOMe for 30 min and immunostained for endogenous LAMP1. Shown are representative confocal single-slice images of three independent experiments. Bar; 10 μm.

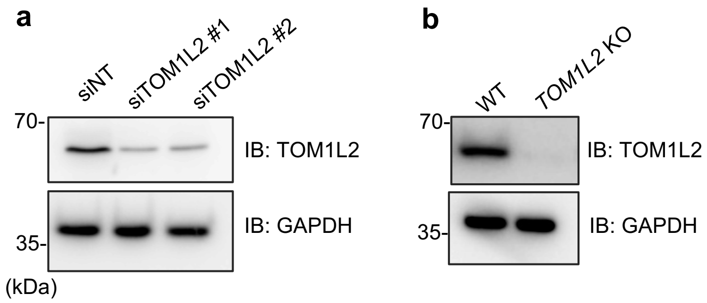

Supplementary Fig. 10 Expression of TOM1L2 in TOM1L2-knockdown or knockout cells.
a, b Immunoblot analysis of TOM1L2 in HeLa cells transfected siRNAs (a) or TOM1L2-knockout cells (b). Shown are representative immunoblot images of three independent experiments. Source data are provided as a Source Data file.
