# Supplementary material for: Rab41-mediated ESCRT machinery repairs membrane rupture by a bacterial toxin in xenophagy
Source: Nat Commun. 2023 Oct 6;14:6230. doi: 10.1038/s41467-023-42039-2 (PMC10558455; doi:10.1038/s41467-023-42039-2)
Supplement: Supplementary file 2 — Reporting Summary [file 41467_2023_42039_MOESM2_ESM.pdf]

nature research

Corresponding author(s): Ichiro Nakagawa

Last updated by author(s): Sep 19, 2023

Reporting Summary

Nature Research wishes to improve the reproducibility of the work that we publish. This form provides structure for consistency and transparency in reporting. For further information on Nature Research policies, see Authors & Referees and the Editorial Policy Checklist .

Statistics

For all statistical analyses, confirm that the following items are present in the figure legend, table legend, main text, or Methods section.

n/a	Confirmed	
☐	☒	The exact sample size (n) for each experimental group/condition, given as a discrete number and unit of measurement
☐	☒	A statement on whether measurements were taken from distinct samples or whether the same sample was measured repeatedly
☐	☒	The statistical test(s) used AND whether they are one- or two-sided<br>*Only common tests should be described solely by name; describe more complex techniques in the Methods section.*
☒	☐	A description of all covariates tested
☒	☐	A description of any assumptions or corrections, such as tests of normality and adjustment for multiple comparisons
☐	☒	A full description of the statistical parameters including central tendency (e.g. means) or other basic estimates (e.g. regression coefficient) AND variation (e.g. standard deviation) or associated estimates of uncertainty (e.g. confidence intervals)
☐	☒	For null hypothesis testing, the test statistic (e.g. F, t, r) with confidence intervals, effect sizes, degrees of freedom and P value noted<br>*Give P values as exact values whenever suitable.*
☒	☐	For Bayesian analysis, information on the choice of priors and Markov chain Monte Carlo settings
☒	☐	For hierarchical and complex designs, identification of the appropriate level for tests and full reporting of outcomes
☐	☒	Estimates of effect sizes (e.g. Cohen's d, Pearson's r), indicating how they were calculated

Our web collection on statistics for biologists contains articles on many of the points above.

Software and code

Policy information about availability of computer code

Data collection	Confocal microscopy data were acquired by Zeiss Zen or Olympus FV1000.
Data analysis	LC-MS data analysis was done using ProteinPilot version 5.0.1 and Progenesis QI version 4.2. Confocal microscopy data analysis was done using Zeiss Zen software, Olympus FV1000 software or ImageJ 1.52 (NIH). All other data analyses were performed using GraphPad Prism 8 (GraphPad Software).

For manuscripts utilizing custom algorithms or software that are central to the research but not yet described in published literature, software must be made available to editors/reviewers. We strongly encourage code deposition in a community repository (e.g. GitHub). See the Nature Research guidelines for submitting code & software for further information.

Data

Policy information about availability of data

All manuscripts must include a data availability statement. This statement should provide the following information, where applicable:

- Accession codes, unique identifiers, or web links for publicly available datasets
- A list of figures that have associated raw data
- A description of any restrictions on data availability

All data that support the findings of this study are available from the corresponding author upon reasonable request.

Field-specific reporting

Please select the one below that is the best fit for your research. If you are not sure, read the appropriate sections before making your selection.

Life sciences study design

All studies must disclose on these points even when the disclosure is negative.

Sample size	No statistical method was used to predetermine sample size. Number of analyzed cells or measurements were chosen to equal or exceed typical standards of the field.
Data exclusions	No data was excluded.
Replication	All results were reproduced at least three times. All attempt for replication were successful and all experiments can be reproduced.
Randomization	Experiments were not randomized, but independent cultures or passages were used for each independent repeat and done on different days.
Blinding	Investigators were not blinded during data acquisition. Analysis of bands or fluorescence intensities was performed by software. But, investigation of the recruitment of proteins to specific targets such as bacteria or autophagosomes were manually counted.

Reporting for specific materials, systems and methods

We require information from authors about some types of materials, experimental systems and methods used in many studies. Here, indicate whether each material, system or method listed is relevant to your study. If you are not sure if a list item applies to your research, read the appropriate section before selecting a response.

Materials & experimental systems

n/a	Involved in the study	
☐	☒	Antibodies
☐	☒	Eukaryotic cell lines
☒	☐	Palaeontology
☒	☐	Animals and other organisms
☒	☐	Human research participants
☒	☐	Clinical data

Methods

n/a	Involved in the study	
☒	☐	ChIP-seq
☒	☐	Flow cytometry
☒	☐	MRI-based neuroimaging

Antibodies

Antibodies used

LAMP1 (H4A3; sc-20011; Santa Cruz Biotechnology, 1:100 for IF)
CHMP4B (13683; Proteintech Group, 1:200 or IF)
CHMP6 (16278-1-AP; Proteintech Group, 1:100 for IF)
VPS28 (15478-1-AP; Proteintech Group, 1:100 for IF)
SNF8 (67696-1-IG; Proteintech Group, 1:100 for IF)
VPS4A/B (17673-1-AP, Proteintech Group, 1:100 for IF)
LC3 (ab51520, Abcam, 1:100 for IF)
LGALS3 (556904, BD Pharmingen, 1:100 for IF)
TOM1L2 (GTX106295; GENETEX, 1:100 for IF, 1:1000 for IB)
streptolysin O (SLO) (ab188539; Abcam, 1:1000 for IB)
HA (HA124; Nacalai Tesque, 1:1000 for IB)
FLAG (M2; A2220; Sigma-Aldrich, 1:1000 for IB)
GFP (GF200; 04363-24; Nacalai Tesque, 1:1000 for IB)
MBP (E8032; New England BioLabs, 1:1000)
GAPDH (sc-47724; Santa cruz biotechnology for IB)
Rab8A (D22D8; 6975S; Cell Signaling Technology, 1:1000 for IB)
Rab41 (NBP2-83434; NOVUS Biologicals, 1:1000 for IB)
Rab41 (18818-1-AP; Proteintech Group, 1:500 for IB)

Validation

The antibodies that have been validated by the supplyers for specific proposes were purchased for our experiment.
In addition, for key antibodies in our study (Rab41 and TOM1L2), we validated the antibodies by western blotting for detecting tagged-proteins.

Eukaryotic cell lines

Policy information about cell lines

Cell line source(s)	HeLa cell line was obtained directly from the American Type Culture Collection.
Authentication	Cell lines were not independently authenticated.
Mycoplasma contamination	Cell lines were routinely tested for mycoplasma and were certified to be negative
Commonly misidentified lines (See ICLAC register)	No cell lines used in this study were found in the database of commonly misidentified cell lines.
