# Supplementary material for: Rab41-mediated ESCRT machinery repairs membrane rupture by a bacterial toxin in xenophagy
Source: Nat Commun. 2023 Oct 6;14:6230. doi: 10.1038/s41467-023-42039-2 (PMC10558455; doi:10.1038/s41467-023-42039-2)
Supplement: Supplementary file 3 — Description of Additional Supplementary Files [file 41467_2023_42039_MOESM3_ESM.pdf]

Description of Additional Supplementary Files:

Supplementary Data 1: Identified proteins from mass spectrometry analysis of Rab41 interacting proteins

Supplementary Data 2: List of qPCR primers used in this study
